# The Correlation between *Helicobacter pylori* Immunoglobulin G Seropositivity and Plasma Homocysteine Levels in Adults

**DOI:** 10.1155/2023/7590549

**Published:** 2023-01-20

**Authors:** Jinke Huang, Xuefei Yang, Jiaqi Zhang, Fengyun Wang, Xudong Tang

**Affiliations:** ^1^Department of Gastroenterology, Xiyuan Hospital of China Academy of Chinese Medical Sciences, Beijing, China; ^2^Department of Gastroenterology, Peking University Traditional Chinese Medicine Clinical Medical School (Xiyuan), Beijing, China; ^3^Institute of Digestive Diseases, Xiyuan Hospital of China Academy of Chinese Medical Sciences, Beijing, China

## Abstract

**Objectives:**

*Helicobacter pylori* (*H. pylori*) immunoglobulin G (IgG) seropositivity is prevalent, but its correlation with homocysteine (Hcy), a biomarker of vascular risk events, is unclear. This study is aimed at exploring the correlation of *H. pylori* IgG seropositivity and plasma Hcy levels in adults.

**Methods:**

Data was obtained from the National Health and Nutrition Examination Survey (NHANES) cycle 1999-2000. Hcy was measured by the Abbott homocysteine assay, and *H. pylori* IgG was measured by enzyme-linked immunosorbent assays. The weighted multiple logistic regression analyses with adjustments for potential confounders were conducted. Subgroup analyses stratified by gender, age, and race were performed.

**Results:**

A total of 4029 subjects aged 20-85 years were included. Population prevalence of *H. pylori* IgG seropositivity was 44.7% in the overall population with higher prevalence found in those with older age, Mexican Americans, lower education, and lower household income. Levels of plasma Hcy were not elevated in those with *H. pylori* IgG seropositivity versus seronegativity (*β* -0.120 (-0.438, 0.199) *P* = 0.462). This difference was not significant after stratifying by gender and age. However, in the subgroup analyses stratified by race, a negative correlation between *H. pylori* seropositivity and plasma Hcy levels was observed in Mexican Americans (*β* -0.802 (-1.253, -0.352) *P* < 0.001).

**Conclusions:**

*H. pylori* IgG seropositivity was not associated with plasma Hcy levels in the general population, but there may be a negative correlation in Mexican Americans. These findings provide new insights to advance the research of the link between plasma Hcy levels and stomach health.

## 1. Introduction

*Helicobacter pylori* (*H. pylori*) is a highly successful pathogen colonizing the human stomach. It was estimated that 4.4 billion people worldwide were infected with *H. pylori* in 2015 [1], and the prevalence of *H. pylori* infection was 35.6% in the United States (US) [2]. *H. pylori* infection has been found to inhibit the secretion of gastric acid and induce chronic inflammation of the gastric mucosa, which alters the gastric microenvironment leading to extensive alterations in the gastric microbiota [3, 4]. Thus, *H. pylori* infection was clearly associated with the onset of a range of gastrointestinal and other systemic diseases [5, 6].

Homocysteine (Hcy) is a sulfhydryl-containing amino acid produced by the metabolism of the essential amino acid methionine. It is clear that the increased concentration of Hcy has a diverse set of pathological functions as it elicits toxic effects on all layers of the arterial wall [7]. There is compelling evidence suggesting that Hcy plays a causative role in the formation and progression of vascular disease [7]. Therefore, the identification of risk factors associated with the increased concentration of Hcy has become a key strategy for the prevention of vascular disease [8, 9].

Current evidence on the correlation between *H. pylori* infection and Hcy levels is limited and conflicting [10–18]. Therefore, it is a topic of concern to further explore the correlation between *H. pylori* infection and Hcy levels to provide evidence for the prevention and treatment of vascular disease. In the present study, the correlation between *H. pylori* immunoglobulin G (IgG) seropositivity and plasma Hcy levels was explored through a retrospective analyses of the National Health and Nutrition Examination Survey (NHANES) database.

## 2. Methods

### 2.1. Study Design and Sample

NHANES is a public database that employs a cross-sectional, stratified, multistage probability design to capture nationally representative samples of the civilian, nonhospitalized population. The survey data included information about questionnaires, demographic data, laboratory tests, and physical examinations. The research protocols were approved by the Research Ethics Review Board of National Center for Health Statistics. All subjects involved in the study provided written informed consent. The study design and survey procedures are available online (https://wwwn.cdc.gov/nchs/nhanes/).

**Figure 1:**
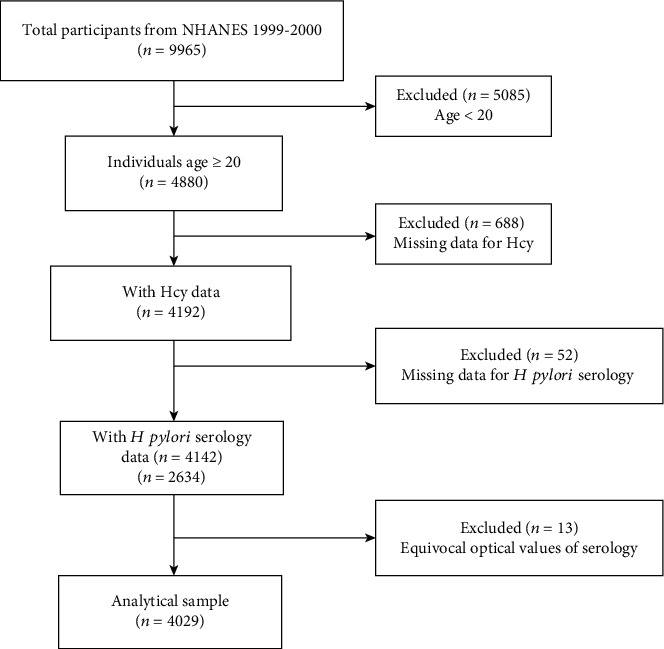
The sample selection flow chart.

NHANES cycles 1999-2000 were selected because *H. pylori* IgG and Hcy were only measured in this cycle. Individuals with information on laboratory and demographic covariates of interest were included in this study, resulting in a final sample size of 4029 adults aged 20 years or older. The participant selection process is shown in the flowchart (Figure 1).

### 2.2. Study Variables

*H. pylori* IgG enzyme-linked immunosorbent assay (ELISA) was conducted on the serum samples of the included subjects. ELISA optical density (OD) values of all subjects were ranging from 0 to 5.73. Participants were classified as *H. pylori* IgG seropositive (OD value ≥ 1.1) or seronegative (OD value < 0.9) according to standard ELISA cutoffs [19]. Subjects with equivocal OD values were excluded to prevent misleading statistical results. Plasma Hcy was measured by the Abbott homocysteine assay. Plasma total Hcy concentrations were calculated by the Abbott IMx Immunoassay Analyzer using a machine-stored calibration curve. For covariates, gender, race, educational level, physical activity, smoking behavior, and other disease status were used as categorical variables; age, poverty to income ratio, days drink in year, serum uric acid, total calcium, blood urea nitrogen, total protein, serum creatinine, total cholesterol, serum vitamin B_12_, and serum folate were used as continuous variables.

### 2.3. Statistical Analyses

The design of complex sampling strategies and appropriate weight were incorporated in all analyses. Weighted multivariate linear regression models were performed to evaluate the correlation between *H. pylori* IgG seropositivity and plasma Hcy levels. The other variables were considered potential confounding factors. Three models were applied to provide statistical inference: model I, no adjustment for covariates; model II, adjusted for gender, age, and race; and model III, adjustment for all covariates. Subgroup analyses stratified by gender, age, and race were performed. These factors were not adjusted when serving as a basis for subgroup analyses. The weighted linear regression model was used to calculate the difference of continuous variables, while the weighted chi-square test was used for categorical variables. All analyses were conducted with EmpowerStats software (http://www.empowwerstats.com, X&Y Solutions, Inc., Boston, MA).

## 3. Results

### 3.1. Characteristics of the Subjects

Population prevalence of *H. pylori* IgG seropositivity was 44.7% in the overall population with higher prevalence found in those with older age, Mexican Americans, lower education, and lower household income. Adults with *H. pylori* IgG seropositive had a higher risk of diabetes and hypertension. More details of the characteristics of the subjects are presented in Table 1.

### 3.2. Correlation of *H. pylori* IgG Seropositive with Plasma Hcy Levels

The results of different multivariate linear regression models are presented in Table 2. In unadjusted model, a positive correlation was found between *H. pylori* IgG seropositive and plasma Hcy levels (1.074 (0.698, 1.449) <0.001). However, after variable adjustments, the correlation between *H. pylori* IgG seropositive and plasma Hcy levels disappeared in model II (*β* -0.268 (-0.568, 0.032) 0.080) or model III (*β* -0.120 (-0.438, 0.199) 0.462).

In the subgroup analyses stratified by gender, no correlation was identified between *H. pylori* IgG seropositive and plasma Hcy levels in either males (*β* -0.230 (-0.754, 0.294) 0.389) or females (*β* -0.064 (-0.399, 0.270) 0.706). Stratifying the analyses by age groups, there was no correlation between *H. pylori* IgG seropositive and plasma Hcy in either age of 20-44 years (*β* 0.016 (-0.329, 0.361) 0.925), 45-59 years (*β* 0.170 (-0.849, 1.189) 0.744), or 60-85 years (*β* -0.178 (-0.726, 0.370) 0.524). Stratifying the analyses by race groups, a negative correlation was identified between *H. pylori* IgG seropositive and plasma Hcy in Mexican American (*β* -0.802 (-1.253, -0.352) <0.001). However, the association was not statistically significant in non-Hispanic white (*β* -0.149 (-0.552, 0.253) 0.468), non-Hispanic black (*β* 0.500 (-0.712, 1.712) 0.420), or other races (*β* 0.426 (-0.456, 1.307) 0.345). Details are presented in Table 2.

## 4. Discussion

The increased concentration of Hcy has been shown to lead to the increased risk of cardiovascular disease and stroke [20, 21]. Endothelial dysfunction is an early event in the development of atherosclerosis, which may be a pathomechanism by which Hcy contributes to the increased risk of vascular disease [22]. Elevated Hcy may affect endothelial function by cytotoxic effects on endothelial cells, stimulating platelet adhesion, or promoting thrombogenic activity [23].

Published studies retrieved from the literature search yielded conflicting results about the correlation between *H. pylori* infection and Hcy levels. The aim of this study was to explore whether there were independent correlations between *H. pylori* IgG seropositive and plasma Hcy levels using the data from NHANES. According to the results of the present study, *H. pylori* IgG seropositivity was not associated with plasma Hcy levels in the general population. However, in the subgroup analyses stratified by race, a negative correlation between *H. pylori* IgG seropositive and plasma Hcy levels was identified in Mexican American. Similarly, some previous studies did not report a correlation between *H. pylori* infection and plasma Hcy levels [15–18]. Two case-control studies [17, 18] of 276 subjects carried out in Iran revealed that there was no significant difference between Hcy in *H. pylori*-infected and *H. pylori*-uninfected participants. However, studies in Japanese populations [11, 15, 16] have reached conflicting conclusions. A case-control study [15] of 90 subjects and a cross-sectional study [16] of 174 subjects revealed that there was no significant difference between Hcy and *H. pylori* infection, while another case-control study [11] which enrolled 93 subjects suggested that *H. pylori*-induced chronic atrophic gastritis decreased plasma vitamin B_12_ and folic acid levels, thus raising homocysteine levels. In Hong Kong, a case-control study of 49 subjects [14] revealed no significant difference between Hcy levels and *H. pylori* infection. Furthermore, no decrease in Hcy levels was found 24 weeks after successful eradication of the bacterium in patients with *H. pylori* infection. Conversely, two studies conducted in Turkey [12, 13] report different results. A case-control study [13] of 86 subjects found that plasma Hcy levels were significantly higher in patients with *H. pylori* infection than in those without *H. pylori* infection. However, another case-control study [12] revealed that the eradication of *H. pylori* helped to decrease Hcy, and the level of Hcy was related to a complex interaction among vitamin B_12_ and folate levels. Alternatively, a retrospective cohort study of healthy Chinese people reported that there was a relationship between *H. pylori* infection and Hcy, and persistent infection resulted in an increase in the latter. In summary, current findings on the correlation between *H. pylori* infection and Hcy levels were limited and conflicting, and the heterogeneity of these studies, including differences in study design, study samples, racial distribution, and controlled confounding variables, may explain the controversial results.

The metabolism of Hcy interacts with folate and vitamin B_12_ [24]. It has been suggested that the increased concentration of Hcy might present in *H. pylori* infection because of the decrement of folic acid and vitamin B_12_ absorption from the diet [25]. However, such logic proposition needs to meet three steps [16]. The first step was that *H. pylori* infection caused gastric atrophy, the second step was that gastric atrophy caused malabsorption to decrease serum folate levels, and the last step was that lower folate levels caused an increase in Hcy levels [16]. Indeed, the first step has been well validated [26]. However, in the second step, few researches have demonstrated that gastric atrophy blocks folate absorption. Conversely, gastric atrophy may not increase the risk of reduced folate levels [18]. The last step was generally recognized, with significantly elevated Hcy concentrations observed in patients with nutritional deficiencies of the essential cofactor vitamin B_12_ and the accessory substrate folate [27, 28]. Furthermore, previous findings suggested that adequate intake of folate and vitamin B_12_ may reduce Hcy levels [29].

To the best of our knowledge, this was the first study to explore the correlation between *H. pylori* IgG seropositive and plasma Hcy levels among US adults. Since a nationally representative sample from NHANES was used, the results of this study were highly relevant to the entire population. However, certain limitations should be acknowledged. First, the data of this study were cross-sectional and therefore cannot be applied to infer causality. Second, although we controlled for many potential confounders (demographics, health behaviors, and clinical status), residual confounding factors might still be present. Furthermore, we preferentially performed subgroup analyses based on the basic demographic characteristics (gender, age, and race), which led to the neglect of other stratification factors that may have a potential impact on the relationship between *H. pylori* IgG seropositivity and plasma Hcy levels, such as body mass index and physical activity.

## 5. Conclusion

*H. pylori* IgG seropositivity was not associated with plasma Hcy levels in the general population, but there may be a negative correlation in Mexican Americans. These findings provide new insights to advance the research of the link between plasma Hcy levels and stomach health.

## Figures and Tables

**Table 1:** Weighted characteristics of the participants.

	*H. pylori* IgG - (*n* = 2228)	*H. pylori* IgG + (*n* = 1801)	*P* value
Age (years)	47.139 ± 18.942	52.919 ± 17.987	<0.001
*Gender (%)*			0.036
Male	45.332	48.640	
Female	54.668	51.360	
*Race (%)*			<0.001
Non-Hispanic white	62.208	24.431	
Non-Hispanic black	14.004	23.209	
Mexican Americans	16.293	40.755	
Other races	7.496	11.605	
*Educational level (%)*			<0.001
Less than high school	23.609	55.691	
High school	25.135	19.822	
College graduate or above	51.257	24.486	
Ratio of family income to poverty	7.030 ± 2.773	5.955 ± 2.537	<0.001
Body mass index	28.242 ± 6.230	28.582 ± 6.119	0.083
*Physical activity (MET-based rank) (100%)*			<0.001
0	22.217	37.146	
1	32.181	30.428	
2	17.415	12.326	
3	28.187	20.100	
*Smoking behavior (%)*			0.042
None	54.354	50.861	
Past	26.571	27.263	
Current	19.075	21.877	
*Diabetes status (%)*			<0.001
None	91.741	85.508	
Yes	8.259	14.492	
*Hypertension status (%)*			<0.001
None	71.140	64.353	
Yes	28.860	35.647	
Days drink in year	80.287 ± 106.027	66.232 ± 100.384	<0.001
Total calcium (mg/dL)	9.398 ± 0.422	9.372 ± 0.410	0.045
Serum uric acid	5.250 ± 1.498	5.375 ± 1.563	0.010
Blood urea nitrogen	14.205 ± 5.874	14.726 ± 5.893	0.005
Total protein	7.473 ± 0.480	7.617 ± 0.481	<0.001
Serum creatinine (mg/dL)	1.559 ± 0.567	1.552 ± 0.579	0.712
Total cholesterol (mg/dL)	198.337 ± 39.934	200.378 ± 41.650	0.114
Hcy (*μ*mol/L)	5.385 ± 0.765	5.519 ± 1.601	0.004
Vitamin B_12_ (pg/mL)	589.824 ± 3165.674	767.302 ± 5430.240	0.196
Serum folate (pg/mL)	16.577 ± 10.886	15.033 ± 9.629	<0.001

Mean ± SD for continuous variables: *P* value was calculated by weighted linear regression model. % for categorical variables: *P* value was calculated by weighted chi-square test.

**Table 2:** Correlation of *H. pylori* IgG seropositive and plasma Hcy levels.

Effect modifier	Model I, *β* (95% CI, *P*)	Model II, *β* (95% CI, *P*)	Model III, *β* (95% CI, *P*)
Total	1.074 (0.698, 1.449) <0.001	-0.268 (-0.568, 0.032) 0.080	-0.120 (-0.438, 0.199) 0.462
*Stratified by gender*			
Male	0.333 (-0.073, 0.740) 0.108	-0.108 (-0.544, 0.329) 0.629	-0.230 (-0.754, 0.294) 0.389
Female	0.397 (-0.014, 0.808) 0.058	-0.406 (-0.817, 0.005) 0.053	-0.064 (-0.399, 0.270) 0.706
*Stratified by age*			
20-44 years	0.152 (-0.127, 0.430) 0.286	0.144 (-0.136, 0.424) 0.313	0.016 (-0.329, 0.361) 0.925
~59 years	-0.156 (-0.856, 0.544) 0.662	-0.310 (-1.087, 0.466) 0.434	0.170 (-0.849, 1.189) 0.744
~85 years	-0.094 (-0.681, 0.493) 0.754	-0.275 (-0.897, 0.347) 0.386	-0.178 (-0.726, 0.370) 0.524
*Stratified by race*			
Non-Hispanic white	1.074 (0.698, 1.449) <0.001	0.209 (-0.129, 0.548) 0.226	-0.149 (-0.552, 0.253) 0.468
Non-Hispanic black	0.328 (-0.867, 1.523) 0.591	-0.866 (-2.035, 0.302) 0.147	0.500 (-0.712, 1.712) 0.420
Mexican American	-0.008 (-0.454, 0.437) 0.971	-0.697 (-1.088, -0.307) <0.001	-0.802 (-1.253, -0.352) <0.001
Other races	1.028 (0.296, 1.759) 0.006	0.212 (-0.480, 0.904) 0.549	0.426 (-0.456, 1.307) 0.345

Model I, no adjustment for covariates; model II, adjusted for gender, age, and race; model III, adjustment for all covariates. For subgroup analyses stratified by gender, gender was not adjusted in model II and model III; stratified by age, age was not adjusted in model II and model III; stratified by race, race was not adjusted in model II and model III.

## Data Availability

Publicly available datasets were analyzed in this study. This data can be found here: https://wwwn.cdc.gov/nchs/nhanes.
